# Investigation and Optimization of the SLM and WEDM Processes’ Parameters for the AlSi10Mg-Sintered Part

**DOI:** 10.3390/ma14020410

**Published:** 2021-01-15

**Authors:** Emilia Franczyk, Magdalena Machno, Wojciech Zębala

**Affiliations:** 1 Department of Production Engineering, Faculty of Mechanical, Cracow University of Technology, 31-155 Cracow, Poland; wojciech.zebala@pk.edu.pl; 2 Department of Rail Vehicles and Transport, Faculty of Mechanical, Cracow University of Technology, 31-155 Cracow, Poland; magdalena.machno@pk.edu.pl

**Keywords:** difficult-to-cut material, AlSi10Mg alloy, aluminum alloy, additive manufacturing, selective laser melting, Wire Electrical Discharge Machining

## Abstract

Presented study concerns the issue of processing the AlSi10Mg aluminum alloy with a use of WEDM technology. Two types of samples tested during the experiment were previously produced in SLM and in casting processes. The aim of the research was to determine the dependence of the input parameters of SLM (laser scanning speed) and WEDM (current amplitude) processes on the performance of the WEDM process as well as on the roughness of the cut surfaces. The experiment was carried out on a specially prepared test stand, and the results’ analysis was carried out using the ANOVA (analysis of variance). A strong influence of the WEDM current on the process speed and on the *Ra* and *Rz* roughness parameters of the produced samples was found. The effect of SLM laser scanning speed was not so strong, but it tended to be uniform. On the other hand, the influence of the tested parameters on the WEDM process energy turned out to be insignificant and irregular. It was also found that for the WEDM process a sample made in SLM technology with relatively high laser scanning speed may be a better choice than the cast one. A case study was carried out to optimize the parameters of the tested processes.

## 1. Introduction

Just behind steel, aluminum and its alloys are the second most common material used in industries such as automotive, aerospace, machinery, manufacturing, transportation, and construction. Wide application of these materials is determined by their specific properties such as light weight, resistance to corrosion, and great mechanical properties including excellent strength and stiffness [1,2,3]. One of the most commonly used aluminum alloys is AlSi10Mg, which belongs to the group of hypoeutectic aluminum alloys [4]. In addition to the abovementioned properties, it is characterized by its low density, excellent castability, and high electrical conductivity [4,5]. Moreover, eutectic Al + Si phase, present in AlSi10Mg alloy, affects its ductility and strength but also makes this material difficult to machine. For this reason, it is mainly used in foundries. Currently, parts made of AlSi10Mg alloy are often produced in complex and irregular shapes that are difficult to obtain with the use of conventional technologies, including casting [5,6]. At the same time, efforts are being made to automate production processes, increase their efficiency, and reduce costs.

In order to ensure feasibility of more and more complex projects, additive manufacturing techniques (AM), also known as “3D printing”, are increasingly used. They involve the production of items based on three-dimensional Computer aided design (3D CAD) models and various methods of cumulative layer deposition. Compared to more conventional manufacturing methods, such as casting or powder sintering, AM technologies allow the production of very complex shapes with a relatively high accuracy and structure densification level [7,8,9,10]. One of the most popular AM techniques for processing metallic powder is Selective Laser Melting (SLM) [2,11]. In this process, a 3D model is divided into thin slices and a raw material (which is a metallic powder with a grain size of 20–60 µm [12]), subjected to thermal energy, is melted in order to form a two-dimensional, responding layer with a thickness ranging from 10 to 80 µm [7,13,14,15]. There are many phenomena that may occur during SLM process, e.g., laser radiation, reflection and absorption, heat transfer, powder melting, phase transformation and melt flow within the molten pool (driven by surface tension gradients), evaporation, and material mass transfer. Such phenomena significantly depend on the properties of metallic powder and on the process parameters [7].

SLM technology not only enables direct fabricating of 3D parts with complex geometry, but also improves mechanical properties of objects made of AlSi10Mg alloy. On the other hand, their lower surface quality (higher values of the *Ra* and *Rz* parameters) and decreased dimensional-shape accuracy in comparison to machined parts are significant disadvantages [13,16,17]. Consequently, additional machining operations are used to achieve the surface quality and geometric accuracy of these items equivalent to finishing and/or semi-finishing. It is worth underlining that the SLM-fabricated parts require subtractive finishing machining but without roughing, which reduces a total time duration of the manufacturing process and constitutes an advantage of this technique. Compared to other materials, the aluminum alloy powders are relatively light, with poor flowability, high reflectivity (91%), and high thermal conductivity (146 W/(m∙°K)) [6,18,19]. Moreover, their ductility can be modified by proper heat treatment. As for the AlSi10Mg alloy, the presence of Si element in its composition decreases its melting point and improves weldability, fatigue performance, and resistance to corrosion. Additionally, the magnesium element (Mg) significantly enhances mechanical strength and impact performance through solution heat treatment and aging, without compromising other desirable mechanical properties [18,19]. On the other hand, low laser absorption and rapid heat dissipation cause some difficulties in manufacturing parts made of aluminum alloys with a use of SLM process. In addition, the Al alloys are subjected to oxidation, which contributes to the phenomenon of forming pores in the sintered material structure. The internal porosity depends also on the process parameters such as laser power, scanning speed, and hatch spacing [19,20]. Therefore, there is a need to conduct further research on the process of melting aluminum alloy powders.

For further processing of the components produced with SLM, both conventional (e.g., turning, milling) and nonconventional methods (e.g., Electrical Discharge Machining (EDM) or Electrochemical Machining (ECM)) are used. Naturally, parameters of these following processes should be matched to the specific material being machined. This is due to the specific mechanical and thermophysical properties of a given material and their influence on the machining process [16,21]. In SLM technology, more and more frequent use is made of metallic powders of difficult-to-cut materials (such as nickel-based superalloys and titanium alloys, hardened and high-strength steels, and composite and ceramic materials). As a result, further processing of these items, performed in order to improve the surface quality to a semi-finishing or finishing class, becomes a challenge for both machining and nonconventional processes [13,18,22,23]. Different structure of the material printed with SLM technology may cause additional difficulties in the manufacturing process. When it comes to difficult-to-cut materials, nonconventional machining methods are mainly preferred. One of these such methods, ensuring high shape and dimensional accuracy (less than 5 µm) and satisfactory surface roughness (*Ra* < 0.1 µm), is electrical discharge machining [24,25]. EDM belongs to the group of alternative technologies due to the electrothermal nature of the material removal process, in which the forces between the tool and the workpiece are negligibly small. Mechanical properties of machined material, such as hardness or ductility, do not affect this type of machining [21,25].

Optimization processes are used in order to select the most advantageous machining parameters for a given material. In the case of EDM, they are performed with a use of mathematical models developed on the basis of investigated effects of selected process parameters (such as current amplitude, pulse-on time, pulse-off time, duty cycle, voltage, capacitance, die-electric flushing pressure, wire feed rate, wire tension) on its performance [26,27,28]. Frequently used optimization techniques include Response Surface Methodology (RSM) [29], Artificial Neural Network (ANN) [30], Taguchi analysis [31], Technique for Order of Preference by Similarity to Ideal Solution (TOPSIS), Gray Relational Analysis (GRA) [32,33], Taguchi Method-Based Gray Analysis [34], and hybrid methods [35,36]. Simulation tools based on the finite element method can also be used for this purpose [37]. Input parameters of EDM process may affect its various outputs such as material removal rate (MRR), tool wear rate (TWR), surface roughness (*Ra* and *Rz*), radial overcut (ROC), crater size, corner deviation, cutting speed, and width [25,27,29,38,39]. Optimization of the EDM process parameters should ensure high MRR while maintaining low levels of TWR, surface roughness, white layer thickness, and surface cracks. In this kind of process, increasing the MRR is usually associated with a deterioration in surface roughness and accelerated tool wear (even up to a 100% in the case of micro-EDM) [24,29].

Many studies published so far concern the optimization of machining processes, including the selection of process parameters affecting its efficiency [29]. However, few of them concern the simultaneous optimization of both processes, the SLM and the additional semi-finishing and/or finishing. An analysis of the influence of SLM laser scanning speed *v* and milling parameters, feed rate (*f*) and milling width (*a_e_*) on the surface roughness (*Ra* and *Rz*), is presented in [18]. RSM method is used to optimize the parameters, and the sintered material is made of AlSi10Mg aluminium powder. The best surface quality (*Ra* = 0.142 ± 0.013 µm and *Rz* = 1.043 ± 0.094 µm) was obtained for the lowest applied parameter values, i.e., *v* = 600 mm/s, *f* = 835 mm/min, and *a_e_* = 0.829 mm. Moreover, the article presents further optimization of the processes with regard to the required parameter values: *Ra* ≤ 0.2 µm and *Rz* ≤ 1.4 µm as well as *Ra* ≤ 0.28 µm and *Rz* ≤ 1.8 µm. Milling of the SLM-made surface also improved its roughness over 20 times. Moving on, the authors of [13] analyzed the effect of longitudinal turning parameters such as cutting speed (*v_c_*), feed (*f*), depth of cut (*a_p_*), and insert corner radius (*r_ε_*) on *Fc* and *Ff* components of the cutting force and on 2D and 3D surface roughness parameters. A workpiece made of AlSi10Mg powder using Direct Metal Laser Sintering (DMLS) was used in the research. The analysis of the obtained results proves that the values of *Ra* and *Rz* depend on the feed rate and corner radius. Moreover, the best result for *Ra* (0.64 µm) was obtained for the cutting parameters of *f* = 0.058 mm/rev, *v_c_* = 300 m/min, *a_p_* = 1.0 mm, and *r_ε_* = 0.4 mm, while the best result for *Rz* (4.33 mm) was for *f* = 0.058 mm/rev, *v_c_* = 200 m/min, *a_p_* = 0.5 mm, and *r_ε_* = 0.2 mm. The Authors also developed an algorithm for selecting the feed value depending on the maximum diameter of the turned shaft but, as they indicate, it needs to be verified when used for other laser-sintered material. Anyways, authors of the above papers did not analyze how the SLM process parameters influence the selection of the finishing process ones. There were also no attempts to optimize in this respect.

Due to the fact that AM technologies are relatively new, there are no optimized procedures for the further treatment of their products in order to improve the surface quality (obtain lower values of *Ra* and *Rz* parameters) or process efficiency. This applies to both conventional and nonconventional manufacturing technologies. Also, for the AlSi10Mg aluminum alloy processed with a use of SLM technology, there were no solutions prepared that would be used to determine the optimal machining parameters regarding surface quality and dimensional accuracy. Selecting the appropriate values of SLM printing parameters dedicated to a specific machining process can make it much easier and faster. Accordingly, the present article focused on the simultaneous study of the effect of laser scanning speed (the SLM process parameter) and the current amplitude, a parameter of Wire Electrical Discharge Machining (WEDM, a type of EDM process). In the first stage of work, samples were made of AlSi10Mg powder using SLM technology and with different values of laser scanning speed. Then they were cut with WEDM using different values of current amplitude. An analysis of the influence of WEDM current amplitude and SLM laser scanning speed on output parameters such as WEDM cutting speed and process energy as well as surface roughness (*Ra* and *Rz*) was carried out. Then, based on the obtained mathematical models, optimal parameter values were established. Additionally, in order to compare the results, WEDM cutting of a cast sample made of AlSi10Mg aluminum alloy was performed using the same current amplitude values.

## 2. Materials and Methods

### 2.1. Workpiece Material and the Tool Electrode Material

Experimental studies on the WEDM process included processing of a sample that was previously produced from the AlSi10Mg metallic powder (m4p material solutions GmbH, Magdeburg, Germany) with the use of the SLM technology. The sample was prepared at the Otto von Guericke University in Magdeburg using a TruPrint 1000 metal 3D printer (Trumpf, Ditzingen, Germany), equipped with a 200-W fiber laser. Constant parameters of the SLM process applied are listed in Table 1.

**Figure 1:**
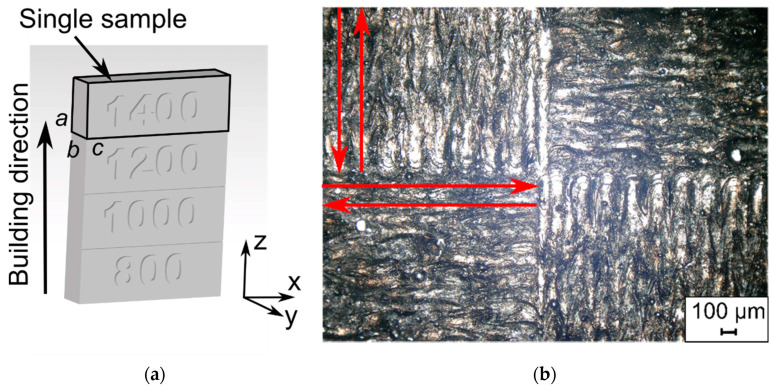
(**a**) The 3D model of specimen; (**b**) a photograph of the applied scanning strategy.

The produced element consisted of four samples with dimensions of a = 7 mm, b = 4 mm, and c = 20 mm, printed one on top of the other, each with a different value of the applied laser scanning speed *v_s_*: 800, 1000, 1200, and 1400 mm/s (Figure 1a). Magnified view of the last sintered layer (*v_s_* = 1400 mm/s) with visible “chessboard” scanning strategy is presented in Figure 1b [40]. Thickness of a single powder layer was ~20 µm.

Additionally, for comparative purposes, the WEDM process was also carried out for a AlSi10Mg cast specimen, the dimensions of which were the same as those of a single sintered sample. Chemical composition of the material of both samples is presented in Table 2.

In the electro-erosion machining process the workpiece material was subjected to thermal stresses and removed as a result of melting, evaporation, and disruption, which were not affected by its toughness. Moreover, free space between electrodes, called the inter-electrode gap, contributed to the fact that the forces occurring in the machining area were negligibly small or absent. However, the presence of high temperature in the machining zone, coming from the plasma channel (10,000 to 20,000 °K), caused the thermophysical properties of the processed material (such as thermal conductivity, density, melting point, evaporation temperature, and a coefficient of thermal expansion) to have a significant impact on the process and its efficiency. Among these properties, thermal conductivity is the one that had the most significant influence on the effectiveness of material removal [21]. In the case of AlSi10Mg alloy, with an increase in temperature from 250 to 750 °K, the thermal conductivity decreased from 2.4 × 10^7^ to 2.0 × 10^7^ erg/(cm·g·°K). After exceeding the temperature of 750 °K, it fell drastically, reaching the value of about 0.90 × 10^7^ erg/(cm·g·°K) at 800 °K. However, with a further increase in temperature, it began to rise again [14]. The density of the 1-inch cube of the AlSi10Mg alloy fabricated via AM was 2.671 + 10.001 g/cm^3^, which was slightly lower than the bulk density of the AlSi10Mg alloy (2.674 g/cm^3^) [9].

An important issue in the EDM process is also the tool electrode material. In the analyzed process, a brass wire with a diameter of 0.25 mm was used as the working electrode. Its thermophysical properties, specified at 973 °K, were as follows: density, 8520 kg/m^3^; thermal conductivity, 110.6 (W/(m·°K)); heat capacity, 385 J/(kg·°K) [41].

### 2.2. Experiment Design

**Figure 2:**
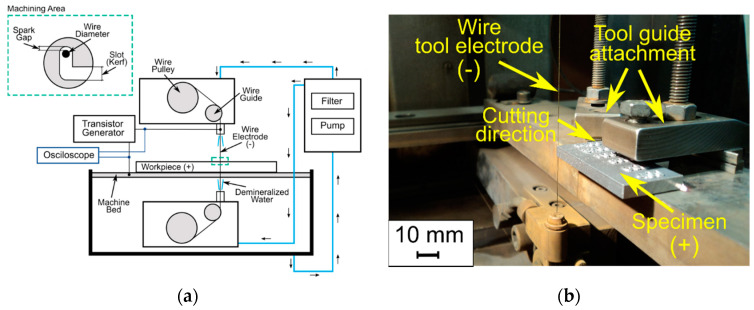
(**a**) Schematic diagram of the test stand and its main functional units; (**b**) a photograph of the experimental setup.

The experimental tests were performed on the electro-erosion wire cutting machine BP95d (Zakład Automatyki Przemysłowej B.P., Końskie, Poland), powered by a transistor generator (Figure 2a,b). Water was supplied to the machining area from above and below with a use of dedicated nozzles. During the tests, the voltage and current waveforms were recorded using the Digital Storage Oscilloscope GDS-1000 series (Rigol, Beijing, China).

During the tests, a slice measuring 1 × 7 × 4 mm was cut from each individual laser-sintered sample. As the samples were printed one on top of the other, the total cutting length was 8 mm (7 mm along the main cutting direction and 1 mm crosswise in order to pull the electrode out of the material between the samples). Cutting process was performed in the direction perpendicular to the layers sintered in the SLM process. In the case of the cast sample, the cutting length was 7 mm.

Machining conditions and parameters of the WEDM process used in this study are listed in Table 3. There were three test repetitions for each set of parameters. During the tests, individual samples were cut with a variable amplitude of current value (in the range of 8–32 A). Fixing the current upper limit at 32 A resulted from the fact that the use of a higher value caused the brass wire electrode to wear out too quickly and, as a result, its breakage just at the beginning of the test. Effective wear of the working electrode depended on many parameters of the EDM process, including open voltage, current amplitude, electrodes’ (tool and workpiece) material, and polarity. Regarding aluminum alloys, their low melting point, located in the range of 843–933 °K, adversely affected the wear of the working electrode [42,43].

The aim of the research was to determine the relationships of the laser scanning speed *v_s_* in the SLM process and the current amplitude *I* in the WEDM process on the roughness parameters *Ra* [µm] and *Rz* [µm] of the cut samples, with the resulting WEDM process parameters: total process energy consumption *E* [kJ] and cutting speed *v* [mm/s]. Research scope also included optimization of the analyzed parameters with regard to their influence on the investigated outputs. The optimization was performed with the use of Response Optimization tool available within the *Minitab* statistical software (Minitab LLC, State College, PA, USA).

Values of surface roughness parameters (*Ra* and *Rz*) were measured using a Taysurf Intra 50 contour measuring tool (Taylor Hobson, Leicester, UK), equipped with a measuring tip with a rounding radius of 2 µm. A measurement speed of 1 mm/s was used. The measurements were performed in the direction transverse to the sintered layers (parallel to the cutting direction). Surface roughness was measured on three elementary sections of operating distance with a length of 1.0 mm. Microscopic observations of the obtained surface were performed with the use of the VHX-600 digital microscope (Keyence, Osaka, Japan) at 500× magnification.

The cutting speed *v* was calculated from the following equation [44]:

*v* = *L*/*t_cutting_*, (1)

where *L* is the cutting length and *t_cutting_* is the cutting time.

The total process energy consumption *E* was calculated from the following equation [45]:

*E* = *U_on_* ∙ *I* ∙ *t_m_*, (2)

where *U_on_* is the relevant open circuit voltage, *I* is the applied current amplitude, and *t_m_* is the time of the WEDM process runtime.

## 3. Results’ Analysis and Discussion

### 3.1. The Impact of Input Parameters on the Cutting Speed and Process Energy Consumption

As a part of the research, regression equation of the WEDM cutting speed *v* (*v_s_*, *I*) (Equation (1)), which is presented below, was determined. Data analysis was performed using the analysis of variance (ANOVA) method. Table 4 presents the ANOVA technique results, where *DF* is degrees of freedom, *Adj SS* is the adjusted sums of squares, and *Adj MS* is the adjusted means squares. The presented equation only consisted of the components for which *p*-value was lower than 0.05.

(3) $$v\;\left(v_{s},\;I\right)=0.30*10^{-5}*v_{s}I+0.91*10^{-4}*I^{2}+0.12*10^{-4}*v_{s}-2.58*10^{-3}*I+1.78*10^{-2}.$$

**Figure 3:**
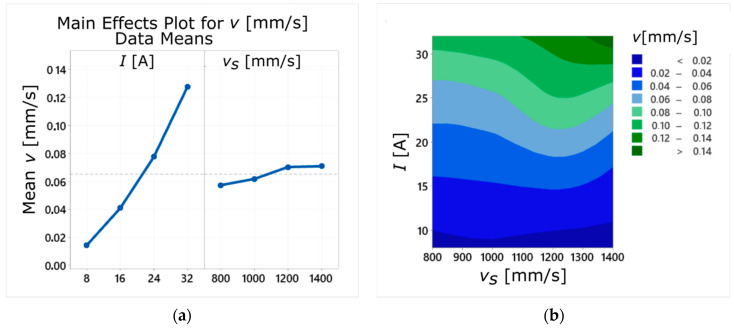
Influence of the process parameters (*I* and *v_s_*) on the values of the cutting speed *v*: (**a**) the main effects’ plot; (**b**) the contour plot.

The influence of variable input factors *v_s_* and *I* on the WEDM cutting speed is shown in Figure 3. According to previous research works on the EDM process, an increase in the current amplitude causes an increase in both the material removal rate and the process energy [25,46]. The analysis of the relationship *v* (*v_s_*, *I*) also showed that increasing the SLM laser scanning speed *v_s_* slightly increased the WEDM cutting speed *v* (Figure 3a). This may indicate that the structure and properties of the sintered material differ depending on the laser scanning speed used, which subsequently affects the material removal rate of the WEDM process. However, the effect of *v_s_* was stronger when higher current amplitude values were used. The reason may be that there were fewer gas pores in the structure of material sintered with *v_s_* = 1400 mm/s, which is important for the EDM process as the electric discharge between electrically conductive surfaces occurs only for a certain thickness of the inter-electrode gap. Presence of gas-filled pores may slow down the electro-erosion process and, thus, the process of removing the material from the workpiece, because it prevents some of the discharges from taking place. Too many irregularly distributed pores can additionally change the thermal conductivity of the sintered material. Relatively high WEDM cutting speed, obtained for the current of *I* = 32 A and *v_s_* = 1400 mm/s (Figure 3b), indicates that in this case the processed material had to be characterized by higher thermal conductivity (improved heat propagation from the so-called surface sources formed on working electrodes under the pressure created during the electro-erosive discharge).

**Figure 4:**
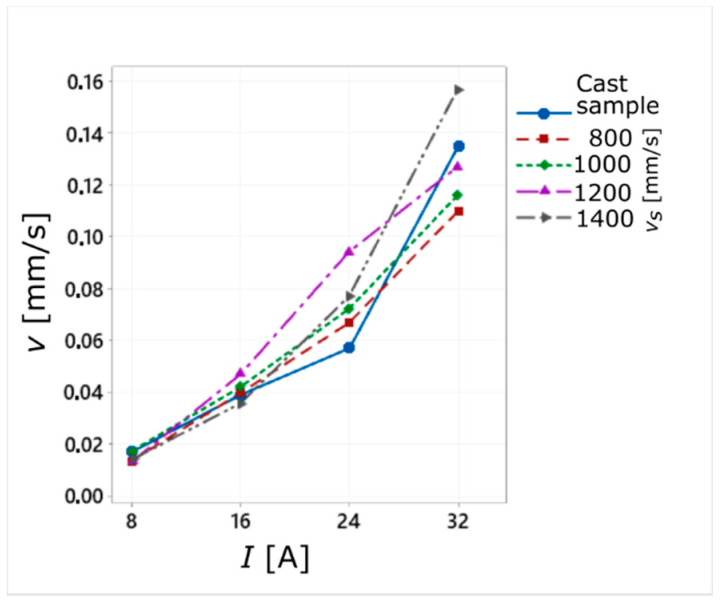
Relationship between input parameters (*I*, *v_s_*) and the average values of the cutting speed *v*.

The above results were compared with those obtained for the cast sample. In this case, the average values of the *v* parameter for the individual values of *I* applied were very similar to the values obtained for the sintered samples (Figure 4). Metallic powder used in the SLM process (AlSi10Mg) has a composition similar to the aluminum alloy applied in the casting process (Table 2), so the melting point and evaporation temperature of the sintered and cast samples should be similar. However, it is worth to notice that the WEDM cutting process achieved the highest speed (*v* = 0.16 mm/s) while the sintered sample was cut with *v_s_* = 1400 mm/s. This observation is significant as many efforts are still being made to increase the material removal rate when using EDM. It turns out it is possible to improve this parameter by processing the material with the same or similar chemical composition, but made using an alternative technology, in this case the SLM method. The conducted research confirms that this solution can be applied for the AlSi10Mg alloy.

**Figure 5:**
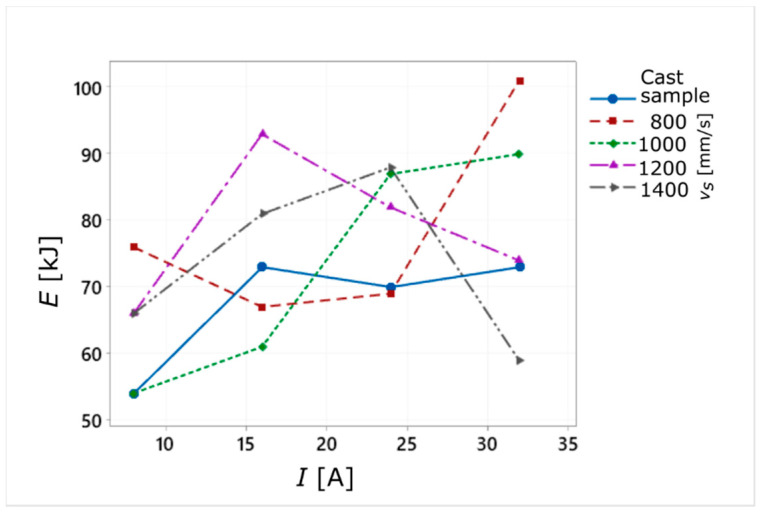
The average values of the total process energy consumption for sintered and cast samples.

On the other hand, the influence of the *v_s_* parameter on the energy of the WEDM process turned out to be insignificant and irregular. The lowest value of the total process energy (*E* = 53.7 kJ) was obtained for *v_s_* = 1000 mm/s and *I* = 8 A, while the highest (*E* = 101.0 kJ) was for *v_s_* = 800 mm/s and *I* = 32 A. In the case of cutting the cast sample, the highest value of process energy was obtained for *I* = 16 A, while the lowest was *I* = 8 A (Figure 5).

### 3.2. The Impact of Input Parameters on the Geometric Structure of Obtained Surface

The ANOVA analysis was also used to determine the effect of the process parameters *v_s_* and *I* on the *Ra* and *Rz* surface roughness parameters for the sintered samples. Regression equations *Ra* and *Rz* (Equation (2) and Equation (3), respectively) were determined taking into account only the significant coefficients (with *p*-value smaller than 0.05). The results of the ANOVA analysis for *Ra* (*v_s_*, *I*) and *Rz* (*v_s_*, *I*) are presented in Table 5 and Table 6.

(4) $$Ra\;\left(v_{s},\;I\right)=-1.1*10^{-4}*v_{s}I+8.14*10^{-3}*v_{s}+22.77*10^{-2}*I-3.37,$$

(5) $$Rz\;\left(v_{s},\;I\right)=6.9*10^{-3}*v_{s}+1.14*I+6.9.$$

**Figure 6:**
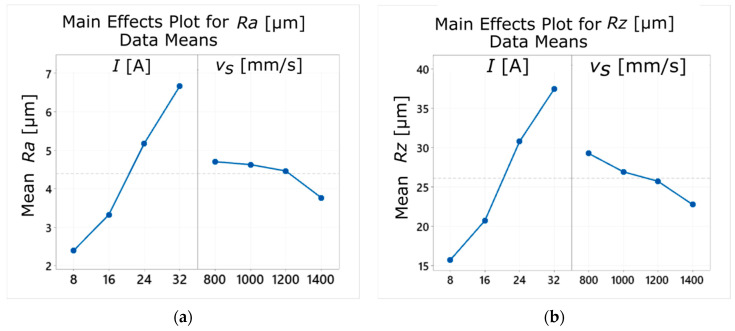
Influence of the process parameters (*I* and *v_s_*) on the values of surface roughness parameters: (**a**) *Ra* and (**b**) *Rz*.

**Figure 7:**
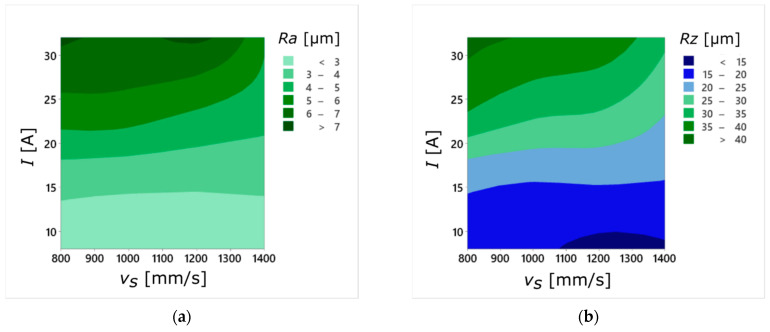
Relationship between the current amplitude (*I*) and the laser scanning speed (*v_s_*) and the average values of surface roughness parameters: (**a**) *Ra*; (**b**) *Rz*.

The experimental research carried out for the sintered sample showed a clear effect of the current amplitude on the surface roughness parameters *Ra* and *Rz* (Figure 6a,b). With the increase of the applied current value, their values also increased (Figure 7a,b). This tendency is consistent with the results of other studies on surface quality obtained in the EDM process [46,47]. It is explained by the fact that applying a higher current value results in the formation of larger erosion craters on the machined surface, as larger amounts of material are then removed during a single discharge. Moreover, it has been found that the laser scanning speed of the SLM process (*v_s_*) also affects the roughness parameters *Ra* and *Rz*. The increase in the *v_s_* value causes the decrease of surface roughness. The lowest mean values of *Rz* (13.6–14.2 µm) were obtained for *v_s_* = 1200–1400 mm/s and *I* = 8 A (Figure 7b).

**Figure 8:**
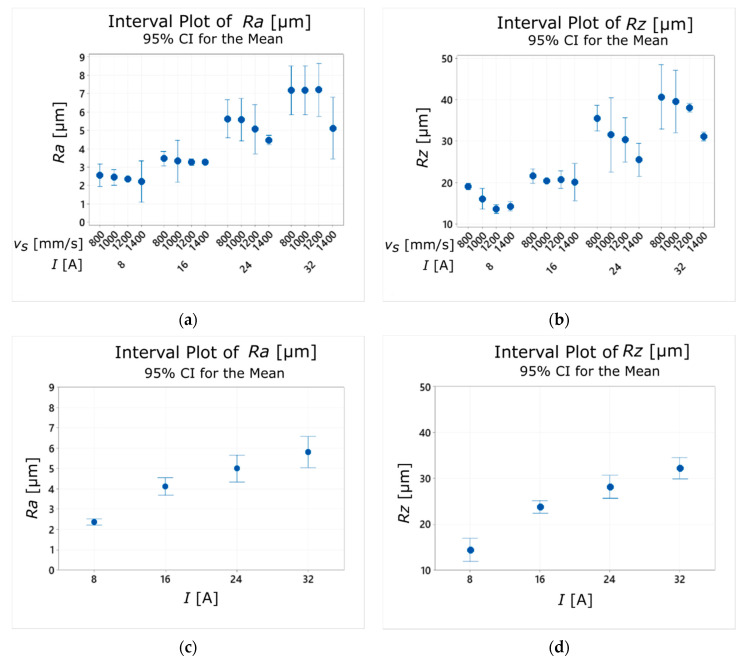
Relationship between input parameters (*I*, *v_s_*) and the average values of surface roughness parameters *Ra* and *Rz* for the sintered sample (**a**,**b**) and the cast sample (**c**,**d**).

The summarized results of surface roughness measurements for the sintered sample (Figure 8a,b) show a clear decrease in the values of the *Ra* and *Rz* parameters with the increase of the laser scanning speed *v_s_*. This dependence occurred for every value of current amplitude used during the WEDM cutting process. Moreover, the mean values of *Ra* and *Rz* obtained for the sample sintered with *v_s_* = 1400 mm/s had similar values to those obtained for the cast sample (Figure 8a–d), regardless of the *I* value.

**Figure 9:**
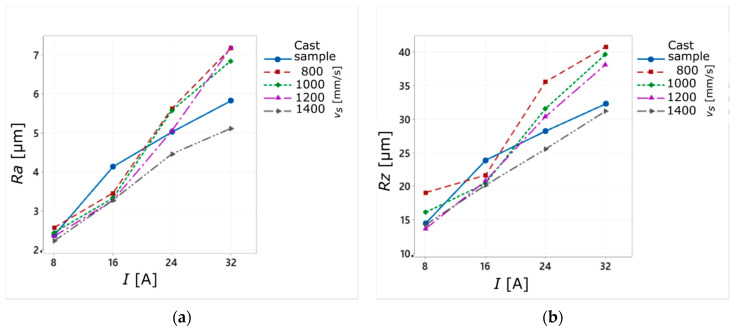
Relationship between the current amplitude (*I*) and the laser scanning speed (*v_s_*) and the average values of surface roughness parameters for the sintered sample and the cast sample: (**a**) *Ra*; (**b**) *Rz*.

A sample manufactured using the SLM technology was formed layer by layer. Increasing laser sintering speed may cause the phenomenon in which the next layer will be applied on the previous one thar has not yet cooled completely, thus raising its temperature again. As a result of this action, the temperature of the sintered layer may again exceed the melting point, which will cause it to melt again. This phenomenon changes the properties of the produced sample, as the individual layers can be more firmly joined together. As a result, the sintered sample may have similar properties to the cast one. On the graphs below (Figure 9a,b), one can see that the *Ra* (*I*) and *Rz* (*I*) plots for the material sintered with *v_s_* = 1400 mm/s and for the cast material are of a similar nature. Also, the *Ra* and *Rz* values for the tested current amplitude values are similar for both types of specimens. It is worth underlining that the average values of those parameters for the sintered material were about 10% lower when compared to the cast sample.

The result of above comparison may constitute a premise to use the AlSi10Mg SLM-sintered workpieces in the WEDM processes. In such a case, the use of higher current values results in obtaining a relatively low surface roughness (compared to the WEDM cutting of the cast material). It also increases the efficiency of the WEDM process, which is particularly important as the low material removal rate is one of its main disadvantages.

**Figure 10:**
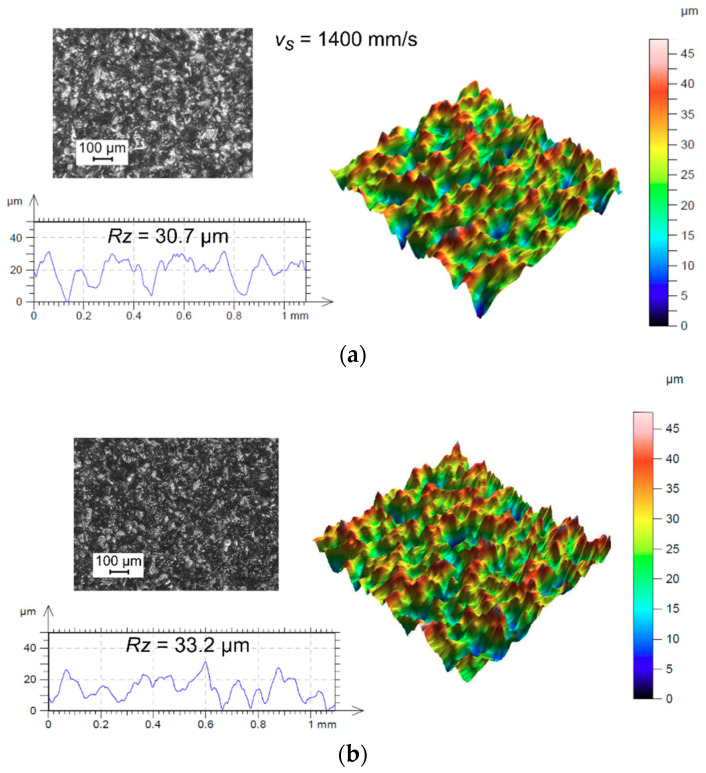
The surface texture in the case of the applied current amplitude if *I* = 32 A for: the sintered sample (**a**) and the cast sample (**b**).

Moreover, both types of samples (sintered and cast) cut in the WEDM process have surfaces of a very similar structure, regardless of the applied current amplitude value. The results of measurements of the surface structures of the sintered sample (*v_s_* = 1400 mm/s) (Figure 10a) and the cast sample (Figure 10b) obtained in the WEDM process with the use of *I* = 32 A are presented below.

### 3.3. The Optimization of Process Parameters with the Use of MiniTab Response Optimizer Tool

**Figure 11:**
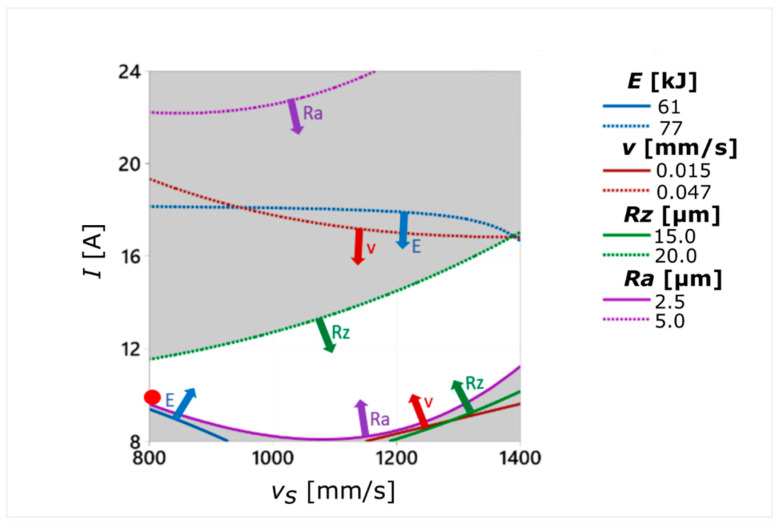
Diagram for selecting the optimum *I* and *v_s_* process parameters.

After finding the relationship between the input and the output parameters of the SLM and WEDM processes, an attempt was made to optimize them within a case study. Based on the assumed values of surface roughness parameters, which corresponded to those obtained in the finishing processes (*Ra* = 2.5–5.0 µm; *Rz* = 15–20 µm), the area of the desired values of variables *I* and *v_s_* was determined. For the surface finish criteria adopted in this way, the values of total process energy and WEDM cutting speed may occur in the following ranges: *E* = 61–77 kJ, *v* = 0.015–0.047 mm/s (Figure 11). The optimal values of the input parameters were selected using the Response Optimizer tool, available within the *MiniTab* software. This method consists of determination of an individual desirability for each response using the assigned weights and boundaries. Obtained values are then combined to determine the overall desirability of the multi-response system. In order to perform the calculations, the reduced gradient algorithm with multiple starting points is used. An optimal solution (input variable setting) occurs when the desirability value obtains its maximum. In the case of the study conducted, the WEDM process energy (*E*) was a factor of utmost importance as it significantly affected the process cost and its environmental impact. In turn, the lowest weight was assigned to the WEDM process speed as the differences in the experimentally obtained results for this parameter were relatively small and did not significantly affect the process runtime. Low efficiency is a specific disadvantage of the WEDM process and still requires improvement. The values of the process variables *I* and *v_s_* were limited to the range investigated within the experiment. The optimal values of the process parameters turned out to be *I* = 9 A and *v_s_* = 800 mm/s. The optimization results and the weights assigned to the individual output parameters are summarized in Table 7. The area marked in white color corresponds to roughness parameter values comparable to the values obtained in the finishing process. The red point represents the parameter setting obtained in the optimization process (Figure 11).

## 4. Conclusions

As a result of the conducted research, it is possible to demonstrate the influence of the tested parameters of the SLM and WEDM processes (laser scanning speed and current amplitude, respectively) on the machined surface roughness and the resulting characteristics of the WEDM process. The following conclusions were obtained on the basis of results analysis:

(1) For the current amplitude of *I* = 32 A, an increase in the laser scanning speed *v_s_* positively affected the cutting speed *v*. Changing the *v_s_* parameter value from 800 to 1400 mm/s resulted in an increase in the cutting speed by approx. 45%, and the value obtained in this case was the highest in the entire research (*v* = 0.16 mm/s).
(2) For the sintered samples, regardless of the applied current amplitude, an increase in the *v_s_* parameter value resulted in an improvement in the surface roughness parameters (decrease in *Ra* and *Rz* parameters values). Moreover, values of these parameters for the sample sintered with *v_s_* = 1400 mm/s were, on average, 10% lower than for the cast sample.
(3) The conducted research showed that during processing of a workpiece made of AlSi10Mg alloy in the SLM technology with sufficiently high laser scanning speed it was possible, in contrast to the cast sample, to increase the WEDM cutting speed and simultaneously to obtain reduced roughness of the machined surface.

In designing the machining process the goal is often to reduce the surface roughness of the manufactured items (although there are exceptions) and to minimize the amount of energy needed to complete the task. On the other hand, its speed, due to the desired high production efficiency, should be as high as possible. Such differentiated criteria imply the need to optimize the process. In the conducted case study, the ranges of process parameters *I* and *v_s_* needed to produce a sample, the quality of which would correspond to the finishing (*Ra* = 2.5–5.0 µm; *Rz* = 15–20 µm), were determined. Then, the multi-criteria optimization process was applied, as a result of which the most favorable parameters of the analyzed processes were selected (*I* = 9 A, *v_s_* = 800 mm/s). For such values of input parameters, the amount of total energy needed to carry out the cutting process was 60 kJ, which was about 60% of the maximum value obtained during the experiment. Due to the relatively low value of the process energy, it was possible to reduce machining costs and to extend the tool electrode life. In this case, the EDM cutting speed reached the value of 0.02 mm/s, which was 1.5 times higher than the lowest value obtained during the experiment.

Further research should focus on determining the impact of other input parameters of the WEDM process (operating voltage, pulse duration and pause time, material of the working electrode) and their interaction with the SLM laser scanning speed on the tested outputs. This would allow for a wider and more accurate study of the machinability of the AlSi10Mg-sintered material regarding WEDM process.

## Figures and Tables

**Table 1:** The SLM process parameters.

Parameter	Value
Laser power [W]	175
Hatching space [μm]	200
Shielding gas	Ar
Diameter of laser beam [μm]	30
Laser wavelength [nm]	1.070
Chamber temperature [°K]	299.95 (27% RF)
Oxygen level [%]	0.11

**Table 2:** The chemical composition of the AlSi10Mg alloy of specimens [wt. %].

Type of Sample	Fe	Si	Mg	Mn	Cu	Ti	Zn	Pb	Sn	Ni	Al
Sintered	0.14	10.6	0.40	<0.01							Ballance
Cast	0.31	9.74	0.20	0.44	0.01	<0.01					Ballance

**Table 3:** Machining conditions for the WEDM process.

Machining Parameter	Value/Characteristic
Current amplitude, *I* [A]	8; 16; 24; 32
Pulse time, *t_on_* [µs]	10
Pulse off time, *t_off_* [µs]	350
The initial interelectrode gap size, *S*_0_ [mm]	0.28
Wire feed rate, *v_f_* [mm/s]	10
Working fluid	Demineralized water with electrical conductivity 89.5 µS/cm
Temperature of working fluid, *T* [°K]	~294

**Table 4:** ANOVA for the cutting speed (*v*). Significant regression coefficients are listed in bold typeface.

Source	*DF*	*Adj SS*	*Adj MS*	*F-*Value	*p-*Value
Model	5	0.029901	0.00598	94.69	0.000
Linear	2	0.028648	0.014324	226.81	0.000
***v_s_***	**1**	**0.000479**	**0.000479**	**7.59**	**0.020**
***I***	**1**	**0.028168**	**0.028168**	**446.02**	**0.000**
Square	2	0.00056	0.00028	4.44	0.042
*v_s_* ^2^	1	0.000015	0.000015	0.24	0.637
***I*^2^**	**1**	**0.000545**	**0.000545**	**8.64**	**0.015**
2-Way Interaction	1	0.000693	0.000693	10.97	0.008
*v_s_* * *I*	**1**	**0.000693**	**0.000693**	**10.97**	**0.008**
Error	10	0.000632	0.000063		
Total	15	0.030532			

**Table 5:** ANOVA for the surface roughness (*Ra*). Significant regression coefficients are listed in bold typeface.

Source	*DF*	*Adj SS*	*Adj MS*	*F-*Value	*p-*Value
Model	5	44.507	8.9014	57.51	0.000
Linear	2	43.2318	21.6159	139.65	0.000
***v_s_***	**1**	**1.6781**	**1.6781**	**10.84**	**0.008**
***I***	**1**	**41.5536**	**41.5536**	**268.45**	**0.000**
Square	2	0.5076	0.2538	1.64	0.242
*v_s_* ^2^	1	0.2898	0.2898	1.87	0.201
*I* ^2^	1	0.2178	0.2178	1.41	0.263
2-Way Interaction	1	0.7677	0.7677	4.96	0.050
*v_s_* * *I*	**1**	**0.7677**	**0.7677**	**4.96**	**0.050**
Error	10	1.5479	0.1548		
Total	15	46.0549			

**Table 6:** ANOVA for the surface roughness (*Rz*). Significant regression coefficients are listed in bold typeface.

Source	*DF*	*Adj SS*	*Adj MS*	*F-*Value	*p-*Value
Model	5	1228.67	245.73	57.41	0.000
Linear	2	1214.02	607.01	141.83	0.000
***v_s_***	**1**	**85.39**	**85.39**	**19.95**	**0.001**
***I***	**1**	**1128.63**	**1128.63**	**263.7**	**0.000**
Square	2	3.2	1.6	0.37	0.697
*v_s_* ^2^	1	0.41	0.41	0.09	0.764
*I* ^2^	1	2.79	2.79	0.65	0.438
2-Way Interaction	1	11.45	11.45	2.68	0.133
*v_s_* * *I*	1	11.45	11.45	2.68	0.133
Error	10	42.8	4.28		
Total	15	1271.47			

**Table 7:** Criteria, parameters, and optimization results.

Parameter	Goal	Weight	Final Value
*Ra* [µm]	Minimize	0.6	2.4
*Rz* [µm]	Minimize	0.6	17.7
*E* [kJ]	Minimize	1	60
*v* [mm/s]	Maximize	0.4	0.02

## Data Availability

The data presented in this study are available on request from the corresponding author. The data are not publicly available due to privacy.
